# The current leadership development opportunities provided for student paramedics by Higher Education Institutions: a literature review

**DOI:** 10.29045/14784726.2020.09.5.2.26

**Published:** 2020-09-01

**Authors:** Alison Rae, Simon Robinson

**Affiliations:** University of Greenwich; London Ambulance Service

**Keywords:** leadership, opportunities, paramedic

## Abstract

**Introduction:**

The development of safe, competent and capable paramedics is one of the key concerns of education providers or Higher Education Institutions (HEIs). To achieve this, paramedic programmes need to focus on teaching leadership to students. The aim of this literature review was to identify the current leadership development opportunities for paramedic students during their undergraduate training across the United Kingdom, in order to identify current gaps and make suggestions on how HEIs could increase leadership opportunities for student paramedics.

**Methods:**

During August 2018, the Scopus, Medline, CINAHL and Academic Search Premier databases were searched (the last three accessed via EBSCOhost). Grey literature was also manually reviewed. Both authors screened the title and abstract and agreed on final papers eligible for full-text review. CASP and COREQ checklists were used to assist in critically appraising the quality of the research and to help decide on the papers chosen for inclusion.

**Results:**

The search yielded 511 results (455 after duplicates were removed). The grey literature search also yielded one additional document that incorporated a framework based on primary research integrated within the paper itself. After title and abstract review, seven papers were included for full text critical review. Two papers were then excluded, resulting in a total of five papers being included in the review.

**Conclusion:**

Current evidence, although limited, demonstrates the benefit of educational programmes in developing educational and non-educational leadership opportunities for paramedic students. Moreover, there is value to individuals being provided or seeking extra-curricular activities, and students should be encouraged to engage in societies, the College of Paramedics, events and conferences, and to work or volunteer in healthcare or emergency service-related sectors to further enhance their leadership potential and skills.

## Introduction and background

Over the past 40 years, the role of the paramedic has developed at a rapid rate. The clinical scope of practice for paramedics in the United Kingdom (UK) now focuses on critical decision making, management and treatment of patients. As such, the development of safe, competent and capable paramedics is one of the key concerns of education providers and Higher Education Institutions (HEIs). Although there are various theories and frameworks that have helped to shape good leadership practice within healthcare organisations over recent years, it is vital that it always contributes to maintaining patient safety (World Health Organisation (WHO), 2009). Therefore, HEIs need to focus on teaching good communication skills, teamwork and safe leadership to students (College of Paramedics, 2015).

Some HEIs already provide a standalone leadership module as part of their paramedic science undergraduate programme, with learning outcomes that are adopted from the Healthcare Leadership Model (National Health Service (NHS), 2013). These modules include objectives such as developing personal leadership potential, understanding the role of leadership in multi-professional team settings and application of leadership theory to paramedic practice. However, there is variability in the type of leadership development opportunities provided by HEIs in the UK (O’Brien et al., 2013; O’Meara et al., 2015). For example, one university prospectus states that leadership is taught in the third year along with teamwork and transitions (University of East Anglia, 2019). Another university states that leadership is taught alongside practice education in the third year (University of Lincoln, 2019). Thus, it is thought that HEIs provide variable modes of opportunities for students to develop leadership attributes, which are not currently consistent throughout the UK. Health Education England (HEE) (2019) states that leadership learning in pre-registration healthcare curricula needs to be explicitly designed so that students have a very clear understanding of how to be effective leaders in practice.

As improving leadership was one of the key themes identified as part of the Francis Report (Department of Health & Francis, 2013), it is an important priority to ensure that all student paramedics being taught within the UK are given ample opportunities to develop their leadership skills. While it is acknowledged that a leadership gap still exists in healthcare (Institute for Public Policy Research (IPPR), 2018), it is further implied that organisations such as the NHS Leadership Academy and the Faculty for Medical Leadership and Management are struggling to nurture a generation of new leaders within the NHS, which is clearly a concern moving forwards. Furthermore, the recent review in ambulance service operational productivity and performance by Carter (NHS, 2018) highlights that there is variance in leadership between services, which directly affects workforce engagement and motivation.

Without enough opportunities to foster and develop clinical leaders, and reduce the inconsistencies of ambulance service leadership, NHS Trusts are at risk of repeating mistakes from the past, hindering progress. It is agreed that healthcare leadership development should begin at undergraduate level (McKimm & Swanwick, 2011; Middleton, 2013; Quince et al., 2014). Therefore, it is important to determine if HEIs are currently providing enough leadership development opportunities to paramedic students or if improvements could be made to the curriculum/programmes.

## Aim

The aim of this literature review was to identify the current leadership development opportunities for paramedic students during their undergraduate training across the United Kingdom in order to identify current gaps and make suggestions on how HEIs could increase leadership opportunities for student paramedics.

## Methods

An integrative literature review was conducted in order to establish and conceptualise the existing research literature based on current student paramedic leadership development opportunities. While the aim focuses on UK-based HEIs and student paramedics, the authors decided that it was pertinent to explore leadership opportunities on a global scale in order to ascertain potential themes that could be introduced to UK student paramedic education.

The Scopus, Medline, CINHAL and Academic Search Premier databases were searched, with the last three being accessed via EBSCOhost. Several searches were conducted during August 2018. For each of the databases, a keyword filter for paramedic literature was applied (Olaussen et al., 2017), consisting of Ambulances OR Emergency Medical Technicians OR Air Ambulances OR paramedic* OR ems OR emt OR prehospital OR pre-hospital OR first responder* OR emergency medical technicians OR emergency services OR Ambulance* OR HEMS OR field triage; and combined with a PICO framework to establish further keywords (Huang et al., 2006) (see Table 1). Opportunities as a keyword was removed due to initial limited search results (n = 55), increasing the risk that the search term may be excluding suitable studies that could be better identified during the screening process.

**Table 1.** PICO (adapted from Huang et al., 2006).

Population	Student paramedics: Keywords: Student* OR undergraduates OR vocation* OR education
Intervention	leadership OR leadership styles OR leadership development
Comparison	None
Outcome	Opportunities – keyword removed in order to broaden the scope of the literature search

Inclusion criteria were: original research that was peer-reviewed and published within the last 5 years; published in English; articles that described student or trainee leadership, and opportunities within educational or placement settings. Exclusion criteria were: editorials, opinion pieces and non-peer-reviewed articles that focused solely on other healthcare professions or registered paramedics. The grey literature was also manually searched using Google Scholar, which included UK government and policy organisations such as Health Education England, the Nuffield Trust and the King’s Fund.

A PRISMA method was used to identify eligible articles (Moher et al., 2009). Both authors then screened the title and abstract, where after comparing results it was unanimously agreed that six papers were eligible for critical review. The authors discussed the action they would take if they could not agree on the suitability of papers. They decided they would ask a colleague with experience in leadership to give their opinion; ultimately, though, this action was not necessary. Critical Appraisal Skills Programme (CASP) (2017) and COREQ (Tong et al., 2007) checklists were used during full text review to assist in critically appraising the quality of the research.

## Results

**Figure 1:**
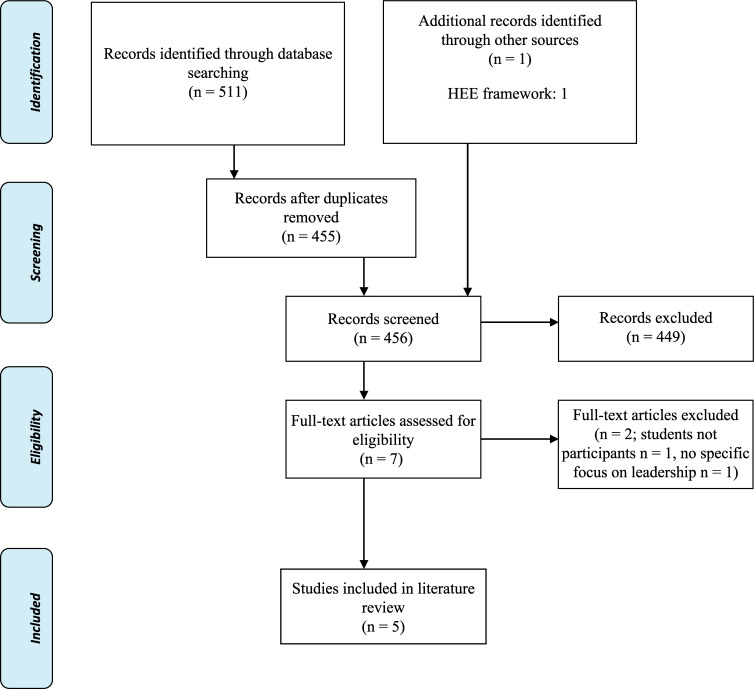
Figure 1. PRISMA flow diagram (Moher et al., 2009).

The database search yielded 511 results (455 after duplicates were removed). After title and abstract review, six papers were included in the full text critical review. After independently completing these critical reviews, both authors concurred that two papers out of the six did not actually meet the inclusion criteria for the following reasons: one did not utilise student paramedics within the study (Brandstorp et al., 2016), and the other did not specifically focus on leadership (Annear et al., 2016). The grey literature search yielded one additional document that incorporated a framework based on primary research integrated within the paper itself, bringing the total to five papers (Figure 1).

Two clear themes were identified for discussion: both educational and non-education opportunities for leadership development and training for pre-registration paramedic students.

Importantly, only one article reviewed pertains to paramedic training in the UK. This was because there was a distinct lack of UK-based research found through the literature search process exploring this area.

The works of Leggio (2014), Mantha et al. (2016) and HEE (2018) explored how healthcare professionals, including paramedic students, learned leadership either through qualitative enquiry (HEE, 2018; Leggio, 2014) – by interviewing participants on their experience of learning leadership – or through post-course leadership surveys (Mantha et al., 2016). The theme identified by the authors was educational, as much of the results focused on classroom learning, educational structure and clinical placement mentoring.

Conversely, Hickson et al. (2014) and Miller (2014) qualitatively examined the role and characteristics of student paramedics themselves. This was also done within Leggio’s (2014) study. Emergent topics centred on previous career experience, as well as activities external to formal education, including attending conferences, sports and volunteering. Subsequently, it was decided to group these elements into the theme of non-educational opportunities. An overview of each study appraised and analysed is listed in Table 2.

**Table 2.** Findings from studies.

Study	Title	Method	Time frame	Sample size	Investigative measures	Relevant findings and themes
**Hickson et al. (2014)**	Engaging in community conversation: A means to improving the paramedicine student clinical placement experience	Qualitative Australia	3-day leadership conference	Two enquiry learning-based groups Three conversation groups	Issues and solutions in relation to the quality in student paramedic clinical placements	Paramedic students attend conferences as much as professionals in order to develop professionalism The student as leader of the group empowered them to adopt a leadership role and engage in discussions in improving clinical placement
**Leggio (2014)**	The state of leadership education in emergency medical services: A multi-national qualitative study	Phenomenological analysis United States, Kingdom of Saudi Arabia, Germany, Philippines and Jordan	June 2013	19 interviews	Learn how EMS providers learned leadership as students and throughout their careers	Primary opportunities for acquiring leadership were identified as: mentoring, on-the-job training, other professions (including previous careers) and courses
**Mantha et al. (2016)**	Adaptive leadership curriculum for Indian paramedic trainees	Follow-up survey comparison India	6-day leadership course	40 paramedic trainees	Investigate a 1-week leadership course strengthening on-scene leadership, teamwork and public speaking skills	Students provided with the opportunity to self-assess might help demonstrate to the individual the benefits of leadership education Combining both lectures and breakout sessions provided more opportunity to develop leadership skills in the classroom
**Miller (2014)**	Exploring paramedic student leadership characteristics in Emergency Medical Services education programs: A grounded theory study	Grounded theory USA	6 weeks in March 2014	35 paramedic students	Understand leadership concepts, characteristics and influential factors from the perspective of paramedic students	Non-educational opportunities for leadership development were categorised as: activities, education (e.g. being involved with academic projects), people, employment and previous career experience
**HEE (2018)**	Maximizing leadership learning in the pre-registration healthcare curricula	Model and Guidelines for Healthcare Education Providers: 2018	2018	27 nurses (inc. six students), eight midwives (inc. five students), 16 physiotherapists (inc. five students), six occupational therapists (inc. three students), one paramedic and one student dietitian	Three universities in the West Midlands, in collaboration with Heath Education West Midlands, undertook a research project to explore leadership development at pre-registration level in non-medical healthcare curricula via three approaches	The main themes analysed included the branding of leadership, responsibility to lead, service user collaboration, inter-professional learning, personal profiling opportunities and assessment in practice, feedback and reflectivity, with recommendations for developing future healthcare leaders Further research and investigation needed in the pre-registration arena

## Discussion

This literature review has determined that there is a lack of research exploring leadership training opportunities for student paramedics within the UK, and that university curricula regarding leadership development are variable and inconsistent. Some HEIs provide standalone leadership modules, while others do not. Furthermore, some HEIs offer a variety of clinical placement opportunities, while others focus on ambulance placements only. Therefore, the overall results of this literature review have determined that current leadership development opportunities provided to student paramedics in the UK are not sufficient and could be improved.

The findings of this review were not unexpected. The benefits of students being afforded both educational and non-educational leadership opportunities over the duration of their undergraduate programme have been clearly shown.

### Educational opportunities

The literature has determined that the paramedic curriculum must incorporate leadership training, but it is not clear in which year of study this training should take place. Currently, it would appear that not all students undertake a specific leadership training module during their programme. This may be because leadership training is incorporated into various modules across the programme rather than being in one specific module. However, Catterall (2018) advises that successful completion of a specific leadership module on a paramedic pre-registration programme enables students to enhance their leadership competence in pre-hospital care. Conversely, Leggio (2014), in his study investigating how leadership is learned in Emergency Medical Services (EMS) in the Kingdom of Saudi Arabia, found that participants did not undertake leadership courses at all during their EMS education. Some students felt that having a specific leadership course as part of their programme would have been very valuable. They also suggested that leadership could be provided as a single course or a progression of two or three courses. Another recommendation that participants made was the provision of simulated patient cases, where a student could lead a scenario in a classroom environment and be given direct feedback by a tutor in order to develop their team-building and leadership skills. Situation debriefing delivered in this manner further supports the enhancement of patient safety initiatives, as discussed by WHO (2009), as it enables students to learn from any errors of judgement they might make, while practising in a safe environment.

HEE (2019) has designed a national framework for simulation-based education which has patient care and safety at its heart. In simulation training, students are expected to react to problems or clinical conditions as they would under genuine circumstances (Munshi, 2015). Power et al. (2013) explored simulation training for paramedics in Ireland. Interestingly, they recognised that the scenarios were normally of low fidelity, thus creating a low level of realism. However, the study only focused on simulated scenarios where the patient required advanced airway management or intravenous administration of medication and fluids, requiring students to make clinical decisions which would arguably draw on existing leadership skills.

Furthermore, HEE (2018) has also produced a model that aims to maximise leadership learning in the pre-registration healthcare curriculum. The model has been developed from a production of workstreams and previous research. It suggests a three-stage approach to developing students as leaders in the pre-registration environment. As a result, HEIs have recently been tasked with reviewing their programmes to ensure development of these stages, including focus on self, working with others and improving healthcare. This ultimately means that there will be changes to the paramedic curriculums over the coming months.

Currently, paramedic practice educators (PPeds) observe, monitor and mentor student leadership development in clinical practice. It could be argued that too much reliance is placed on the PPed or practice mentor to build the links between leadership theory and practice (Myall et al., 2008). Seemingly, the PPed theory–practice mentorship and module approach means that certain students may fail to truly understand the relevance of leadership, possibly due to the variation in PPEds’ level of knowledge regarding recent educational and programme reforms (Givati et al., 2018; Willis et al., 2010).

Mantha et al. (2016) studied the implementation of a 6-day leadership curriculum for 40 paramedic trainees in the first academic year of a 2-year advanced post-graduate degree in emergency care in India. Students were asked to deliver a video-recorded presentation on a randomly selected but simple topic before and after the course. Each trainee was only given 2 minutes to prepare their topic before presenting to their classmates. Independent judges reported a significant improvement in confidence, posture, presentation gestures and stage confidence in the presentations delivered after the course. Being confident is an important trait for being an effective leader, as it promotes self-assurance, belief in success and accomplishment of goals (Northouse, 2015). This concept is further supported by the College of Paramedics (2017), which states that paramedics must be able to demonstrate confidence in practice.

### Non-educational opportunities

The literature demonstrated that leadership opportunities for paramedic students extend beyond the clinical practice setting and educational environment. For example, Miller’s (2014) thesis, which interviewed 35 North American paramedic students, highlighted three key non-educational leadership opportunities. These were: 1) activities, such as athletics, sports and societies, that enabled students to formulate their concepts of leadership; 2) people, including teachers, academic staff, friends and family, and the influences they have on leadership, e.g. one participant stated that her leadership had developed by virtue of being a mother; 3) employment and previous career experience, such as a managerial role or working as a healthcare assistant in a different healthcare setting. These findings indicate that students who seek to improve their leadership skills should become involved in university societies and gain further experience of working in a supervisory role within a healthcare setting. Indeed, Foli et al. (2014) found that American nursing students involved in designing and implementing a health fair at university significantly improved in leadership behaviours.

Furthermore, Hickson et al. (2014) observed 53 delegates at a 3-day Australian leadership conference, including 30 paramedics and 16 paramedic students, engage in ‘community conversations’ about key issues surrounding clinical placements. The student paramedics were given the lead roles within the group, and this was found to empower them to feel included in serious conversations and to enhance their experience of adopting a leadership role, steering professional development. Conferences, therefore, should encourage students to adopt an active role in either organisation or participation. In the UK, movements such as Project A (Association of Ambulance Chief Executives (AACE), 2018) offer students the opportunity to become ambassadors, leading in developing ideas for improving ambulance services. While Project A is government funded, however, attending other conferences can be costly and some students may not wish to participate due to the financial strain it might cause.

The College of Paramedics supports extracurricular activities by recommending students attend annual conferences and events pertinent to their roles. Indeed, students are encouraged to be involved within the college itself, such as organising local events or representing the student body. Leggio (2014) reported that leadership opportunities in other professions were available to students, such as volunteering with the military, or working and training with other services such as police and fire. Volunteering in such settings might offer an improved understanding of inter-professional working, further enhancing leadership development. Miller (2014) supports this concept further, with his study additionally noting that paramedic students acknowledged the importance of leadership as a collaborative process – students felt that being involved with a team reduced the perspective of hierarchical leadership.

### Limitations

Primarily, the literature review was limited by the fact that research on leadership development for student paramedics is severely lacking, especially in the UK. Broadening the scope to include registered paramedics may have yielded a more thorough understanding of how leadership develops and could have helped to draw conclusions that could be brought into higher education.

Furthermore, the review method would have yielded better reliability by using a more systematic approach. For example, a more systematic search of the grey literature may have resulted in more studies. The majority of the research appraised did not focus on paramedic leadership practice within the UK. Therefore the findings of this review may not be generalisable to UK HEIs and paramedic programmes. Additionally, the studies found from the literature review focus on different types of leadership theory and clinical settings, rendering it difficult to compare and contrast these findings. Accordingly, this review took an integrative stance in order to pave the way for future research to focus on developing a more evidence-based approach to delivering leadership opportunities to paramedic students in the UK.

### Recommendations

A comparative study is needed to explore the various leadership opportunities currently provided to paramedic students by all HEIs providing paramedic training programmes in the UK. A further qualitative research study that explores students’ views on leadership training opportunities would also help to strengthen the following conclusions.

## Conclusion

This literature review has determined that HEIs should focus on incorporating leadership theory and development into relevant core modules over at least 2 years of the undergraduate programme. This is supported by HEE (2018), which suggests that leadership learning should be embedded into programme design. HEIs should also consider the benefits provided by low- and high-fidelity simulation training, which supports practice-based learning using clinical scenarios that require leadership skills as part of clinical decision making. Furthermore, HEIs need to organise a variety of clinical placements, exposing students to a diverse range of multi-professional working across the NHS. The Health Care Professional Council (HCPC) (2018) supports this further by acknowledging the benefit of learners working with other relevant professionals, including nurses. Finally, HEIs must encourage students to attend conferences, join societies, become ambassadors and volunteer for other organisations, which will give them additional opportunities to demonstrate leadership, while at the same time encouraging them to collaborate with other disciplines.

## Author contributions

AR wrote the Introduction and background, Aim, Discussion, Educational Opportunities, Recommendations and Conclusion sections. She is the key author. She also made all of the final changes leading to acceptance of the review for publication. SR completed the PRISMA diagram, as well as the Methods, Results and Non-educational opportunities sections. AR acts as the guarantor for this article.

## Conflict of interest

None declared.

## Funding

None.
